# Impact of early social housing on the play behavior of neonatal and post-weaning dairy calves

**DOI:** 10.3389/fvets.2025.1683861

**Published:** 2025-09-08

**Authors:** Ciara McKay, Kathryn Ellis, Marie J. Haskell, Nicola Gladden

**Affiliations:** ^1^School of Biodiversity, One Health and Veterinary Medicine, University of Glasgow, Glasgow, United Kingdom; ^2^Scotland's Rural College (SRUC), Edinburgh, United Kingdom; ^3^School of Veterinary Medicine and Science, University of Nottingham, Nottingham, United Kingdom

**Keywords:** dairy calf behavior, positive animal welfare, play behavior, social housing, calf housing

## Abstract

We aimed to assess the impact of early life housing and play experiences on neonatal and weaned calves play behavior. A total of 96 female dairy calves were recruited from four Scottish dairy farms and assigned to individual (*n* = 48), paired (*n* = 24) or group (*n* = 24) housing at birth. Play behavior was measured using IceTag accelerometers (Peacock Technology, UK) during two experimental periods in the same cohort of calves, at neonatal and weaned stages. A mixed-effects negative binomial regression analysis was used to assess how early social housing influences the play behavior of neonatal and weaned calves. The analysis also considered the impact of early play on the play behavior of weaned calves. Calves housed in paired or group pens from birth performed significantly more neonatal play compared to calves housed individually from birth. No lasting effect of early life housing on weaned calf play behavior was observed. There was no correlation between counts of neonatal and weaned calf play. Calves with lower neonatal playfulness showed a numerical increase in play behavior after weaning compared to those with higher neonatal playfulness. These findings add to the growing body of literature indicating that early life social housing provides a more positive welfare experience for pre-weaned dairy calves. The study also highlights the need for future research to understand the impact of rearing experiences and different management systems on play behavior in weaned calves.

## 1 Introduction

The early life housing experience of dairy calves impacts their development, health, and welfare. Research has demonstrated the importance of early life socialization of calves and links social housing, where calves are reared in pairs or groups, to improvements in growth, feed intake and cognitive development (1). However, despite the growing body of evidence supporting social housing, individual housing remains the most prevalent type of housing for newborn dairy calves in the United Kingdom (2), the USA (3) and in several European countries (4–6).

Newborn dairy calves have traditionally been housed in individual pens due to the perceived benefits of controlling respiratory and enteric pathogens (4) with health concerns commonly found to be the main limiting factor for producers adopting social housing (2). However, data supporting these hypotheses are conflicting, with some authors reporting an increased risk of diseases including bovine respiratory disease (BRD) and neonatal calf diarrhea (7, 8) in socially housed calves; while others report no difference (9) or even improvements in health parameters in socially housed calves (10). Cross-suckling, where calves suckle on each other, is often seen as a negative behavior associated with paired and group housing (2, 3). However, because it’s driven by hunger and motivation to suckle (11), it may be reduced by increasing milk volumes, using teats instead of open buckets, and providing enrichment like dummy teats or scented hay (12, 13).

Another suggested advantage of individual housing is to allow individualized feeding regimes (14). Some producers cite concern over the monitoring of individual calf feeding habits as a drawback to social housing (2). The growing popularity of automated calf feeders has overcome these concerns as they allow producers to create individualized feed plans and monitoring systems in group-housed calves (15). Moreover, social housing facilitates learning of feeding behaviors and can lead to increased starter feed intakes compared to individually housed calves (16–18). Improved feed consumption in socially housed calves can have a positive impact on growth, and multiple studies have shown increased daily liveweight gain over the pre-weaning period in calves housed in pairs or groups compared to individually housed calves (16, 19).

Social housing can improve cognitive and behavioral development in dairy calves. Pair-housed calves are reported to have more bold and competitive personality traits than individually housed calves (20). Compared with calves reared individually from birth, calves raised in group and pair settings are more accepting of changes to their management including the introduction of new feedstuffs (21), new environments (22) and meeting unfamiliar calves (23). Additionally, calves reared in social settings form bonds with their pen-mates and show a preference to interact with familiar calves over other pen-mates (24, 25). Social bonds formed early in life provide emotional support that may allow calves to better cope with stressful events such as weaning or mixing of social groups (26).

The long-term benefits of early life housing on calf development and the influence of early social experience on adult cattle is a topic of growing interest. When subjected to behavioral testing at 6 months old, calves reared in social settings, either with their dam or with other calves, display more social behaviors toward other calves and have a shorter latency to enter an open arena compared to individually reared calves (27). It has been shown that the social behaviors learned early in life aid calves with the transition into the mature dairy herd, where socially reared calves are more dominant later in life, adapt better to competition in cubicle housing and are more likely to remain in the herd past the first lactation than individually reared calves (28, 29). However, even with these improved adult social skills, studies have failed to find a link between early life socialization and improved production parameters such as fertility or milk yield (29, 30). Despite knowing the short-term welfare benefits and long-term developmental benefits of early life social housing, the lasting impact of early life housing on calf welfare, specifically the impact on play behavior, has not been investigated.

The welfare impact of different calf housing types can be measured using behavioral monitoring. Play behavior is performed by calves when they feel free from stress or threats to their wellbeing and is widely recognized as an indicator of positive animal welfare (31). The role of play in calf development is not fully understood, however it has been suggested that play may be important for building social skills and preparing calves to cope within a mature herd setting (32). Variability in playfulness, both at an individual and group level, may be related to welfare-relevant factors such as health or environmental challenges which negatively influence the animal’s affective state (31). The welfare impact of different calf management systems can be assessed using play, where increased play behavior would indicate that an environment provides a more positive welfare experience. It is widely reported that calves housed socially from birth are more active and display more play behavior than calves housed individually throughout the whole pre-weaning period (10, 33–35). Though several authors have shown the association between social housing and increased play behavior, most studies record play using direct visual behavioral observations which can be labor intensive and are often not practical in an on-farm setting (36). Play can also be monitored using wearable accelerometer devices, and over the past decade, different devices have been validated to measure play in dairy calves of varying ages in the pre- and post-weaning periods (37–41). Despite technological advances to monitor calf play behavior, no studies to date have used direct accelerometer outputs to measure calf play behavior to compare the welfare states across multiple different on-farm early life housing conditions.

Across two sampling periods, we examined the impact of early life housing on neonatal and weaned dairy calf play behavior. In Period 1, leg mounted accelerometer technology was used to measure play behavior in calves across individual, pair and group housing from birth. We hypothesized that calves in social housing types would play more than calves in individual pens. In Period 2, the same leg mounted accelerometer technology was used to measure play behavior in the same cohort of calves post-weaning, to determine if early life housing and early life play experiences influence play in older calves. We hypothesized that calves housed socially pre-weaning would play more post-weaning than individually housed calves and that, regardless of housing type, calves categorized as more playful early in life would remain more playful after weaning.

## 2 Materials and methods

### 2.1 Calf recruitment

A total of 96 Holstein, Friesian and Holstein-Friesian cross calves were recruited at birth from four commercial year-round calving dairy herds in central and southwest Scotland. Only female calves subject to unassisted birth were eligible for recruitment. Data were collected over a 26-month period from March 2022 to May 2024. Farms were recruited based on their current calf housing practices and a willingness to adopt an additional method of housing for a proportion of calves for the duration of the study. Herd size ranged from 170 to 850 milking cows. *A priori* a sample size of 24 calves per farm was selected based on similar previous work comparing play between housing types (33–35) together with practical consideration. For Period 1, 12 neonatal calves per farm were assigned to housing in individual pens (Farms 1, 2, 3 & 4) and 12 neonatal calves per farm were assigned to housing in paired (Farms 1 & 4) or group pens (Farms 2 & 3).

### 2.2 Calf management

Calf management practices varied between farms and are summarized in Table 1. All calves had their umbilici dipped with an iodine solution and were fitted with an identification ear tag immediately after birth, following UK animal identification regulations. On all farms, calves received colostrum within 4–6 hours of birth and were removed from the dam soon after (< 8 h after birth). Two farms housed calves both individually and in pairs, with one farm using straw-bedded calf pens and the other using straw-bedded hutches. The remaining two farms housed calves both individually and in groups in straw-bedded calf pens accommodating four to six calves per group. All housing types met, or exceeded, the minimum standards for housing calves according to the European Council Directive 2008/119/EC. Milk feeding was provided twice daily from a teat or an open bucket. Three farms provided a commercial milk replacer product immediately after colostrum feeding, while one farm provided cow’s transition milk for 3 days before feeding commercial milk replacer (Table 1). Calves on all farms had ad libitum access to water, roughage and concentrates starting from birth. Age of weaning and weaning protocols varied between farms (Table 1). All calves were housed in straw-bedded group pens following weaning.

**Table 1:** Summary of pre- and post-weaning calf housing and nutritional management.

Management characteristic	Farm 1	Farm 2	Farm 3	Farm 4
Milking herd size	430	170	850	240
Calf breed	Holstein	Friesian	Holstein	Holstein Friesian
Calf birthweight (mean; range)	39 kg (28-49 kg)	43 kg (37-51 kg)	39 kg (29-53 kg)	50 kg (38-58 kg)
Colostrum management	4 L via stomach tube	3 L via stomach tube	4 L via bottle	4 L via bottle
Pre-weaning housing	Straw-bedded individual or paired hutches with an outside run from birth to 6 days old	Straw-bedded individual or group pens of 4–5 calves from birth to weaning	Straw-bedded individual or group pens of 5–6 calves from birth to 10 days old	Straw-bedded individual or paired pens from birth to 14 days old
Pre-weaning pen dimensions	Individual: 1.5 m x 1.1 m x 1.2 m hutch & 1.4 m x 1.1 m run Paired: 2.2 m x 1.2 m x 1.5 m hutch & 1.6 m x 1.2 m run	Individual: 1.6 m x 0.95 m Group: 3.1 m x 2.1 m	Individual: 1.5 m x 0.92 m Group: 3.0 m x 3.0 m	Individual: 1.8 m x 1.2 m Paired: 1.8 m x 1.8 m
Pre-weaning space allowance	Individual: 3.19m^2^ /calf (total) Paired: 2.28m^2^ /calf (total)	Individual: 1.52m^2^ /calf Group: 1.30–1.62m^2^ /calf	Individual: 1.38m^2^ /calf Group: 1.50–1.80m^2^ /calf	Individual: 2.16m^2^ /calf Paired: 1.62m^2^ /calf
Milk feeding management	3 L milk replacer twice daily via teat bucket	3 L milk replacer twice daily via open bucket	3 L transition milk twice daily via teat bottle until 3 days old, then 3 L milk replacer twice daily via teat bucket	3 L milk replacer twice daily via open bucket
Milk replacer composition	24% crude protein (whey), 20% crude fat, 0% crude fiber, 7.5% crude ash	24% crude protein (whey), 20% crude fat, 0% crude fiber, 7.5% crude ash	23% crude protein (skim), 25.5% crude fat, < 0.05% crude fiber, 7% crude ash	23% crude protein (whey), 18% crude fat, 0% crude fiber, 7.5% crude ash
Weaning management	7-day milk volume reduction to wean by 56 days old	5-day milk volume reduction to wean by 70 days old	5-day milk volume reduction to wean by 65 days old	5-day milk volume reduction to wean at 60 days old
Post-weaning housing	Igloo with straw-bedded group pen of up to 10 calves	Straw-bedded group pen of up to 10 calves	Straw-bedded group pen of up to 30 calves	Straw-bedded group pen of up to 20 calves
Post-weaning pen dimensions	3.9 m x 4.4 m x 2.2 m igloo & 5.1 m x 5.1 m pen	8.4 m x 4.9 m	5.4 × 18.1 m	14.0 × 10.5 m
Post-weaning space allowance	4.32m^2^ /calf (total)	4.12m^2^ /calf	3.26m^2^ /calf	7.35m^2^ /calf

### 2.3 Period 1: data collection on neonatal calf play behavior

Following birth, calves were assigned by farm staff to a housing group depending on pen availability on the farm. Within 24 h of birth, calves were fitted with a tri-axial accelerometer (IceTag, Peacock Technology Ltd., UK) on the lateral hindlimb following the protocol described by Gladden et al. (39). The data output from this accelerometer, specifically the metric termed “motion index” (MI), which is a measure of overall animal activity, has been validated for measuring play behavior in neonatal dairy calves (39). Motion index is a proprietary metric generated by the IceTag which takes into account the duration and forces applied during movement to provide an overall indication of animal activity (39).

At the time of IceTag application, calves were weighed to establish birth weight using either an electronic scale or weigh band placed around the girth behind the forelimb. Additionally, calves were visually examined by a veterinarian from the Scottish Center for Production Animal Health & Food Safety (SCPAHFS) to assess health status before IceTag application. IceTags were attached to measure neonatal calf play behavior over a 48-h recording period from 24 to 72 h old. In paired pens, IceTags were attached to one calf only, with the other calf in the pair acting as a companion animal. In group pens, IceTags were attached to a maximum of two calves at any one time, with all other calves in the pen acting as companion animals. IceTags were removed at the end of the recording period and the data were downloaded using an IceReader device and IceManager software (Peacock Technology Ltd., UK). Data were output as 1-min sampling intervals and the presence or absence of play in each interval was recorded using a MI threshold of ≥ 3 (39). Complete IceTag datasets were available for 85 out of the 96 recruited calves, as 11 calves had missing data due to the accelerometer malfunction.

### 2.4 Period 2: data collection on weaned calf play behavior

Play behavior was measured in the same cohort of calves following weaning when the calves were aged 3–5 months old (mean 139 d, SD ± 16 d). IceTags were fitted to the lateral hindlimb of each calf for a 48-h recording period following the protocol described by McKay et al. (41). Data collection was performed over a two-week period on each farm individually, with staggered recording periods that meant IceTags were fitted to a maximum of six calves per pen at any one time. Farm medicine records were consulted at the time of IceTag application to record any treatment or disease events, including diarrhea, bovine respiratory disease, bloat and omphalitis, occurring between birth and the second data collection period. IceTags were removed at the end of the recording period and the data were downloaded as for the neonatal calves. Data were output as 15-min sampling intervals and the presence or absence of play in each interval was recorded using a MI threshold of ≥ 69 (41). Complete IceTag datasets were available for 74 out of the 85 calves with full neonatal datasets either due to accelerometer malfunction (*n* = 6) or mortality (*n* = 5).

### 2.5 Ethics statement

Ethical approval for the study was obtained from the University of Glasgow School of Veterinary Medicine Research Ethics Committee (ref EA15/22).

### 2.6 Statistical analysis

Data were organized and summarized in Microsoft Excel (Version 2,411, Microsoft Corporation, USA) and exported to Stata 18 (Release 18, StataCorp LLC, USA) for analysis. Data were examined for normality by the visual appraisal of histograms and the Shapiro Wilks tests of normality. Descriptive statistics were calculated for each recording period (neonatal and weaned) to establish the percentage of IceTag output intervals in which calves were engaged in play. Boxplots were constructed to visually assess the factors influencing counts of neonatal and weaned play.

Cumulative incidence of disease was 33.8% (27.0% [20/74] pneumonia; 1.4% [1/74] diarrhea; 2.7% [2/74] bloat; 2.7% [2/74] omphalitis). Due to a low incidence of individual diseases within the dataset, any recording of disease was dichotomised within a “health event” (yes/no) variable. To establish if calves remain more playful over time, calves were categorized into a “play quartile” based, regardless of housing type, on their count of neonatal play with Q1 calves being the least playful and Q4 calves being the most playful. All other data including housing type, dam parity and farm were analyzed as categorical variables.

To determine if calves housed socially play more than calves housed individually in Period 1, a multilevel mixed effects negative binomial regression model was used to analyze the impact of early life housing on count of neonatal play behavior, using neonatal housing type and dam parity as fixed effects. To determine if early life housing and early life playfulness influence play in older calves during Period 2, a second multilevel mixed effects negative binomial regression model was used to analyze the impact of early life experience on count of weaned play behavior, using neonatal housing type, dam parity, health event and neonatal play quartile as fixed effects. For both models, farm and calf ID were included as random effects, with calf ID nested within farm. Stepwise regression using Akaike Information Criterion (AIC) was conducted to find the best model significance and fit. Post-hoc pairwise comparison was used to assess differences in incidence rate ratio (IRR) for categorical variables with three or more levels. Results were considered statistically significant at *p* ≤ 0.05.

## 3 Results

### 3.1 Period 1: observed influences on neonatal calf play behavior

Regardless of housing type, across the 48-h recording period the mean percentage of all 1-min IceTag intervals in which neonatal calves were found to be playing was 10% (292/2880) and ranged from 4% (110/2880) for the least playful calf, to 22% (635/2880) for the most playful calf. The mean percentage of intervals playing differed between housing types with individually housed calves playing in 9% (247/2880) of intervals, pair-housed calves playing in 11% (322/2880) of intervals and group-housed calves playing in 12% (353/2880) of intervals.

Eighty-five calves were included in the final analysis of neonatal play behavior (Table 2). Compared to calves housed individually, pair-housed calves had a 1.29 times higher rate of play (*p* = 0.002) while group-housed calves had a 1.43 times higher rate of play (*p* < 0.001). *Post hoc* pairwise comparison of group-housed calves versus pair-housed calves found no significant difference in play between the two housing types (IRR = 1.11; *p* = 0.334). There was no difference in neonatal play between calves born to multiparous compared to primiparous dams (IRR = 1.10; *p* = 0.170).

**Table 2:** Final multilevel mixed effects negative binomial regression model of count of neonatal calf play behavior.

Variable	Incidence rate ratio	Standard error	*z*	*p* > |*z*|	95% confidence interval
Early life housing					
Individual	Reference				
Paired	1.29	0.11	3.04	0.002	1.10–1.53
Group	1.43	0.11	4.77	< 0.001	1.24–1.66
Dam parity					
Primiparous	Reference				
Multiparous	1.10	0.08	1.37	0.170	0.96–1.27

### 3.2 Period 2: observed influences on weaned calf play behavior

Regardless of housing type, across the 48-h recording period the mean percentage of 15-min IceTag intervals in which weaned calves were found to be playing was 23% (45/192) and ranged from 5% (10/192) for the least playful calf to 44% (84/192) for the most playful calf. The mean percentage intervals of playing differed between housing types with previously individually housed calves playing in 23% (44/192) of intervals, previously pair-housed calves playing in 26% (49/192) of intervals and previously group-housed calves playing in 23% (44/192) of intervals.

Seventy-four calves were included in the final analysis of weaned play behavior (Table 3). No effect of early life housing on weaned calf play behavior was detected, with no significant difference in weaned play between paired versus individually reared calves (IRR = 1.21; *p* = 0.381); group versus individually reared calves (IRR = 1.03; *p* = 0.789) or group versus pair reared calves (IRR = 0.91; *p* = 0.599). Dam parity also had no significant impact on weaned calf play (IRR = 1.03; *p* = 0.801). The presence of a health event between the two experimental periods (IRR = 1.06; *p* = 0.534) had no significant impact on weaned calf play.

**Table 3:** Final multilevel mixed effects negative binomial regression model of count of weaned calf play behavior.

Variable	Incidence rate ratio	Standard error	*z*	*p* > |*z*|	95% confidence interval
Early life housing					
Individual	Reference				
Paired	1.12	0.15	0.88	0.381	0.87–1.45
Group	1.03	0.12	0.27	0.789	0.82–1.30
Dam parity					
Primiparous	Reference				
Multiparous	1.03	0.11	0.25	0.801	0.84–1.25
Health event					
No	Reference				
Yes	1.06	0.10	0.62	0.534	0.88–1.28
Play quartile					
Q4	Reference				
Q3	1.22	0.16	1.46	0.143	0.94–1.58
Q2	1.15	0.15	1.13	0.260	0.90–1.48
Q1	1.09	0.16	0.59	0.555	0.82–1.45

**Figure 1:**
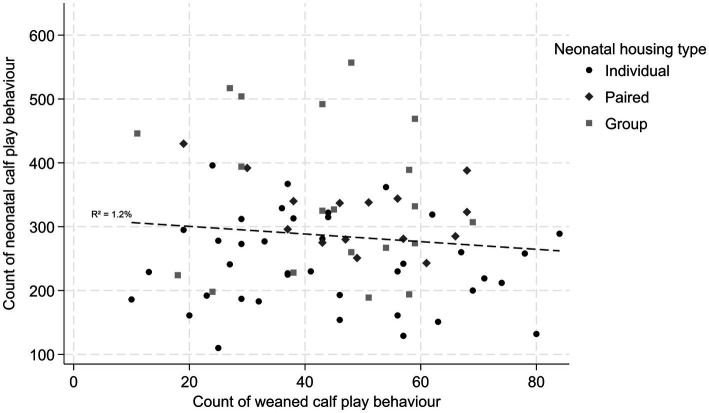
Scatterplot and regression line demonstrating the correlation between count of neonatal and weaned calf play behavior by housing type.

No correlation between the count of neonatal play and the count of weaned play was identified (Figure 1). There was no difference in weaned play between calves in different neonatal play quartiles, though compared to calves in the highest neonatal play quartile (Q4), all calves in the lower quartile of play displayed numerically more play when weaned (Q4 versus Q3 IRR = 1.22; *p* = 0.143; Q4 versus Q2 IRR = 1.15; *p* = 0.260; Q4 versus Q1 IRR = 1.09; *p* = 0.555).

## 4 Discussion

We found that calves housed in either paired or group pens from birth engaged in more neonatal play behavior than calves housed individually. This finding agrees with other studies reporting increased play in calves housed in pairs (34) or groups (33, 35) from birth. Given that play is an indicator of positive animal welfare, our results suggest that social housing from birth provides calves with a better early life experience. Moreover, play is believed to support somatic development, build fitness and enhance physical and emotional skills that may be required later in life (31). The improved play experience observed in socially housed calves may therefore provide them with a longer term developmental advantage over less playful individually housed calves. Interestingly, we found no difference in neonatal play between pair-housed and group-housed calves. Given the known relationship between increased space allowance and increased calf play behavior (42), it is possible that the lower space allowance per calf in the group pens reduced their ability to perform unrestricted play. Alternatively, these findings may indicate that the presence of social companionship, regardless of group size, promotes play and improved calf welfare. Though no previous studies have compared pair and group housing, Lv et al. (35) similarly found no difference in play between calves housed in groups of three, six or twelve. The contagious nature of play, where it becomes more likely for pen-mates to start playing after an individual play bout is started (31), likely contributes to an increase in this behavior and an improvement in affective state in socially housed calves. This finding is important for producers looking to implement social housing, as a housing type can be selected based on factors such as ease of management or space availability since all social housing types, regardless of group size, increase play and improve early life welfare.

Recent work assessing calf vigor in beef suckler herds has shown that calves born from primiparous cows are less vigorous and take more time to stand following birth than calves born from multiparous cows (43). We therefore hypothesized that calves born to primiparous dams may be less active and display less play behavior in the neonatal recording period; however, we found no dam parity effect on neonatal play. This finding may be explained by our calf recruitment protocol where only calves born from unassisted calvings were eligible for inclusion in the study. Primiparous cows are three times more likely to experience dystocia compared to multiparous cows (44) and assisted calving has been associated with reduced play behavior in newborn dairy calves in the first 48 h of life (45). Additionally, primiparous cows display reduced motivation to attend to their offspring immediately after calving which may reduce calf vigor and latency to stand (46) though in our study, calves were separated from the dam immediately after birth so poorer mothering by primiparous animals was less likely to impact calf activity.

The impact of early life housing on calf development is an area of growing interest, and multiple previous studies have found early social housing to have a positive long-term impact on calf behavior. Studies have indicated that personality traits such as boldness or social behaviors such as exploration of novel environments and interaction with new pen-mates are influenced by early life housing, with individually housed calves being characterized as less bold and more reactive to social novelty (23, 47). The negative impact of early life individual housing on calf behavior appears to carry forward and has been shown to negatively impact calves’ adaptation to new environments and social groups at key management stages such as after weaning (48), at 6 months old (27) and when introduced to the adult herd prior to first calving (28). We found no lasting impact of early life housing on play behavior in weaned calves, suggesting that play may not be a learned behavior or personality type which is influenced by early life housing. If play is a behavioral response to positive welfare conditions rather than a developed personality trait, these findings suggest that a reduced early life play experience can be overcome. This finding is particularly important for systems such as those buying in replacement heifers or calf finishing units where calf management is not controlled by the final producer. As play is a dynamic behavior that is highly influenced by current housing and management factors such as space allowance, social contact and food availability (19, 24, 42), producers may potentially reverse poor early life experiences and improve immediate welfare states by providing enriched, comfortable environments to facilitate play after weaning.

While early life play experience is believed to strengthen an animal’s physical and mental capabilities to allow them to perform more play later in life (31), we found no difference in weaned play between calves characterized as more or less playful early in life – a contrast to our original hypothesis. We found that compared to the most playful neonatal calves (Q4), calves characterized as less playful in the neonatal period displayed numerically more play events in the weaned play recording period. Although no significant relationship was found, this difference could be linked to a long-term rebound effect of restricted early life play. While no studies have shown that motivation to play builds over a prolonged period, previous work has described a short-term impact of restricted play: pre-weaned calves deprived of the ability to play in individual pens will build motivation to play and will display increased play behavior compared to socially reared calves when exposed to an environment with ample space allowance (49, 50).

A limitation of our study is the variation in weaned calf housing, particularly space allowance, across the four farms in Period 2. While the effect of space allowance on weaned calf play behavior has not been studied, it is well understood that increased space allowance is associated with increased play in the home pen of pre-weaned dairy calves (42, 51). Low space allowance does not prevent play behavior but restricts the capacity of multiple calves to perform it simultaneously (52). Given that accelerometers were used to measure play in up to six weaned calves during each recording period, it is possible that some calves on the farms with lower space allowance were unable to express play at all times. Additionally, social structure and familiarity of pen mates may have influenced expression of play in the weaned calf recording period as it is known that calves form social bonds early in life which can carry forward to promote more positive social behavioral interactions after weaning (53). Findings regarding the impact of early life experience on weaned calf play are potentially confounded by management and may vary across different farms depending on with different space allowances and social structures. To investigate this relationship further, future work studying a greater number of farms and husbandry systems is warranted.

When assessed using visual observations, calf play behavior has been reported to decline with increasing age (52, 54). Similar effects (comparing play in the neonatal and post-weaning periods) could not be assessed in this study due to the nature of accelerometer data recording which defines only the presence or absence of play within a given period but does not describe the exact count, duration and nature of play. Several authors have reported restrictions in the ability of accelerometer technology to accurately describe the nature of specific calf behaviors including play (39–41, 55). At this time, the use of IceTags to measure play behavior is limited to a research setting as these devices have become commercially unavailable since the completion of this study. An unavoidable limitation of using accelerometer technology as an alternative to visual observation in the study of calf play behavior is the need for validation which is specific to the device and management system studied. Various commercially available accelerometers exist as wearable devices for calves (15), and it is recommended that this technology is validated to measure play in order to be used for real-time, on-farm welfare assessment.

Given the limitations of using accelerometer technology to monitor calf play behavior, it is recommended that future work should also focus on validating alternative forms of commercially available technology for on-fam behavioral analysis and welfare assessment. Recent work by Vázquez-Diosdado et al. (56) used location data provided by ultra-wideband sensors to monitor calf play behavior over 18 weeks. Unlike accelerometers, this technology allowed constant and detailed reporting of play behavior and may be the future of both research-based and on-farm sensor-driven measurement of calf play behavior. Computer vision (CV), which uses cameras and machine learning techniques to analyze the behavior of farm animals, can provide useful insights into calf health and welfare by tracking changes in animal posture, activity and behavioral patterns (57). While this technology is not currently validated to measure play in calves, it may have the potential to offer a fully automated, non-invasive method of measuring play, and thus positive welfare states, in calves.

## 5 Conclusion

This study provides valuable insights into the impact of early life housing and play experiences on the behavior of dairy calves. Our findings show that calves housed in paired or group pens from birth perform more neonatal play than calves housed individually and confirms that social housing from birth should be promoted on farms in order to improve early life welfare. Contrary to our hypothesis, we found no lasting impact of early life housing or early life playfulness on post-weaning play behavior. These findings suggest that the welfare impacts of early life housing, both positive and negative, do not carry forward into later stages of development and that negative early life experiences can potentially be compensated for with improved management conditions after weaning. Although early play experience is believed to strengthen an animal’s future capability to play, our findings suggest that play is more of a dynamic positive behavioral response influenced by current management and environmental factors. The results of our study indicate that future work is required to understand the balance between behavioral development and management factors which influence play behavior in weaned calves. It is recommended to implement social housing from birth in order to improve neonatal calf well-being, with ongoing awareness of the importance of adapting post-weaning management to maintain these benefits.

## Data Availability

The raw data supporting the conclusions of this article will be made available by the authors, without undue reservation.
